# Editorial: RNA modifications and epitranscriptomics, Volume II

**DOI:** 10.3389/fgene.2023.1229046

**Published:** 2023-06-07

**Authors:** Kunqi Chen, Ernesto Picardi, Xiao Han, Giovanni Nigita

**Affiliations:** ^1^Key Laboratory of Gastrointestinal Cancer, Ministry of Education, School of Basic Medical Sciences, Fujian Medical University, Fuzhou, China; ^2^ Department of Biosciences, Biotechnology and Environment, University of Bari Aldo Moro, Bari, Italy; ^3^ College of Biological Science and Engineering, Fuzhou University, Fuzhou, China; ^4^ Department of Cancer Biology and Genetics and Comprehensive Cancer Center, The Ohio State University, Columbus, OH, United States

**Keywords:** RNA methylation, RNA editing, psuedourylation, epitranscriptomics, RNA modification, RNA

RNA modifications have been proven to be an important complement to epigenetics. Currently, at least 170 types of RNA modifications have been identified among all three life domains. In the past decade, several molecular functions of RNA modifications have been unveiled, including RNA structure switches, RNA stability, RNA export, and translation. RNA modification-associated biological processes such as neuro and embryo development, cell cycle, and stress response were also investigated. Additionally, aberrant RNA modifications have been observed in multiple diseases and are considered as potential therapeutic targets. However, compared with well-studied DNA modifications and posttranslational modifications, the biological meanings of RNA modifications have not been yet deciphered in detail. To facilitate the understanding of RNA modifications and their biological roles, we collected 14 articles on this Research Topic, including 13 research articles and a review.

Pecori et al. provide unique work on this Research Topic, focusing on the RNA editing mechanism. Based on high-throughput sequencing methods, the authors found putative U-to-C editing sites. A more in-depth analysis revealed that such sites may be misinterpreted as novel modification events, resulting instead from A-to-I editing on overlapping antisense RNAs that are transcribed from the same loci. Their findings were experimentally validated by RT‒qPCR and editing quantification.

Noncentral nervous system sepsis can cause sepsis-related encephalopathy (SAE), a brain dysfunction disease. To identify the potential biomarkers and association among the gut microbiome, serum metabolomic profile, and RNA m6A methylation in SAE patients, Wang H. et al. collected twenty patients with and without SAE. In this work, authors integrated multiple experimental methods, such as ELISA, RT‒qPCR, 16S rDNA sequencing, and LC-MS/MS. The ELISA and RT‒qPCR results showed positive correlations between IL-6, ICAM-5, and the m6A methyltransferase METTL3, while the m6A demethylase FTO was decreased in SAE patients. Interestingly, a positive correlation between the abundance of *Acinetobacter* and the expression of METTL3 was also observed, which affected the diversity of the gut microbiome. In general, m6A regulators could be used for SAE screening.

Three studies used high-throughput sequencing to show the landscape of posttranscriptional regulation. She et al. provided an epitranscriptome profile in villous tissues from spontaneous abortion (SA). They applied MeRIP-seq to detect methylation regions and integrated bioinformatics analysis. Based on the sequencing results, the authors suggest that the methylation distribution and motifs differ in SA and normal conditions. In the conjoint analysis of meRIP-seq and RNA-seq, the enriched gene ontology and KEGG pathways also differed between SA and normal conditions. Additionally, their results suggested that m6A modification plays an important role in SA by regulating lysine degradation and the Hippo signaling pathway. In summary, the authors believe their findings provide an alternative therapeutic target for spontaneous abortion.

Wang S. et al. presented the epitranscriptome of the mammary gland tissues of dairy goats at different lactation stages. They applied MeRIP-seq to show 2,476 and 1,451 m6A methylation peaks during the early and peak stages of lactation, respectively. The distribution of m6A peaks among transcriptomes differs at the early and peak stages, whereas the motif is similar in different stages. The differentially methylated genes were further analyzed by gene ontology and KEGG pathway analyses, and the results suggest that hypo- or hypermethylated genes participate in biological processes, such as cell apoptosis, cell growth processes, cellular components, or biogenesis. Finally, the hub genes show that HRAS, JUN, and EGFR may play the most important roles in the lactation stages.

miRNA is another type of posttranscriptional regulation. Fei et al. used high-throughput miRNA-seq to analyze differentially expressed miRNAs in the liver tissue between Hu (short/fat-tailed) sheep and Tibetan (short/thin-tailed) sheep. Compared with Hu sheep, six upregulated and five downregulated miRNAs were observed in Tibetan sheep. Miranda and RNAhybrid were used to predict the target of miRNA. The differentially expressed miRNAs and their target genes were integrated into gene ontology and KEGG pathway analysis. In addition to bioinformatics analysis, oar-miR-432-regulated SIRT1 was validated by Western blotting. In general, authors believe their work could provide a theory to study the fat metabolism of sheep.

Two works focused on colon cancer. He et al. provided a bioinformatics analysis to show the potential association between RNA methylation and lncRNAs in colon cancer, which could be biomarkers for hot and cold tumors and prognosis. They used RNA-seq data from the colon cancer cohort from TCGA and identified m1A/m5C/m6A/m7G-related lncRNAs based on Pearson correlation. In a further step, univariate Cox regression analysis was applied to identify 23 RNA modification-related lncRNAs with prognostic value. Additionally, the patients classified into different groups based on RNA modification-related lncRNAs had different clinical characteristics in immune microenvironmental infiltration and immunotherapy response. The authors believe their work will contribute to personalized treatment regimens. Other works presented by Li et al. analyzed the necroptosis-related genes in the colon cancer cohort and the potential association between necroptosis-related genes and RNA modifications.

Gao et al. and Lu et al. showed that m6A and m5C participate in the development of liver disease, respectively. Gao et al. used wet lab methodologies to identify that the m6A methyltransferase METTL16 contributes to liver fibrosis in chronic hepatitis B infection. Lu et al. used bioinformatics analysis to identify m5C-related lncRNAs in hepatocellular carcinoma.

Bioinformatics analysis with experimental verification was integrated into osteoporosis, breast cancer, and uterine fibroids studies. Qiao et al. analyzed high-throughput sequencing data to show that m6A regulators are biomarkers in osteoporosis, which was validated by experiments. Huang et al. used TCGA-BRCA RNA-seq data to identify m7G-related lncRNAs and validated them by RT‒qPCR. Cai et al. analyzed previously published DNA and RNA methylation profiles to study uterine fibroids and validated them by experiments to identify PLP1 as a biomarker. Another work presented by Wang Z. et al. used different datasets to build a prediction model for the prognosis of idiopathic pulmonary fibrosis and validation.

Sun et al. provided a review article to summarize the roles of m6A methylation in aging and aging-associated diseases, including tumors, neurodegenerative diseases, diabetes, and cardiovascular diseases. In addition, the authors also discussed the association between m6A methylation and autophagy, inflammation, oxidative stress, and DNA damage. Considering the importance of m6A in aging and disease development, the authors suggest that m6A-related drugs should be developed to address the challenges of aging.

Generally, in this Research Topic, 13 articles focused on posttranscriptional regulation-related biological processes or disease development covering m1A/m5C/m6A/m7G, and one article studied the mechanism of RNA editing. We hope our Research Topic enhances our understanding of RNA modifications.

